# 3D motion of vesicles along microtubules helps them to circumvent obstacles in cells

**DOI:** 10.1242/jcs.201178

**Published:** 2017-06-01

**Authors:** Ione Verdeny-Vilanova, Fabian Wehnekamp, Nitin Mohan, Ángel Sandoval Álvarez, Joseph Steven Borbely, Jason John Otterstrom, Don C. Lamb, Melike Lakadamyali

**Affiliations:** 1 ICFO-Institut de Ciències Fotòniques, The Barcelona Institute of Science and Technology, Castelldefels, Barcelona 08860, Spain; 2 Ludwig-Maximilians-Universität München, Department of Chemistry, Physical Chemistry, Center for Integrated Protein Science Munich, and Nanosystems Initiative Munich, Butenandtstr. 5-13, München 81377, Germany

**Keywords:** 3D tracking, Motor protein, Super-resolution microscopy, Vesicle trafficking

## Abstract

Vesicle transport is regulated at multiple levels, including regulation by scaffolding proteins and the cytoskeleton. This tight regulation is essential, since slowing or stoppage of transport can cause accumulation of obstacles and has been linked to diseases. Understanding the mechanisms by which transport is regulated as well as how motor proteins overcome obstacles can give important clues as to how these mechanisms break down in disease states. Here, we describe that the cytoskeleton architecture impacts transport in a vesicle-size-dependent manner, leading to pausing of vesicles larger than the separation of the microtubules. We further develop methods capable of following 3D transport processes in living cells. Using these methods, we show that vesicles move using two different modes along the microtubule. Off-axis motion, which leads to repositioning of the vesicle in 3D along the microtubule, correlates with the presence of steric obstacles and may help in circumventing them.

## Abstract (precis)

**Highlighted Article:** We develop a new method to track vesicle dynamics in three dimensions and show that vesicles change their position while actively transporting along microtubules in living cells to overcome obstacles.

## INTRODUCTION

Motor proteins actively transport vesicles by translocating along microtubules (Hirokawa, 1998; Vale, 2003). Intracellular transport is tightly regulated by different mechanisms, such as scaffolding proteins, motor protein clustering and the cytoskeleton (Rai et al., 2016; Fu et al., 2014; Fu and Holzbaur, 2014; Toropova et al., 2014; Zajac et al., 2013; Nirschl et al., 2016), among which, regulation by the local cytoskeletal structure is the least well understood. By using correlative live-cell and super-resolution microscopy (Bálint et al., 2013; Tam et al., 2014a,b), we previously showed that pausing of endolysosomes correlates with tight microtubule intersections (Bálint et al., 2013). However, whether microtubule intersections can act as selective filters, impacting trafficking of certain types of vesicles differentially is currently unknown.

A permanent pausing at microtubule intersections would cause accumulation of obstacles leading to transport failures (De Vos et al., 2008; Kanaan et al., 2013; Staff et al., 2011; Stokin and Goldstein, 2006a,b). In healthy cells, pausing is temporary and vesicles are rapidly cleared from intersections (Bálint et al., 2013). Understanding the mechanisms by which motors circumvent such obstacles can give important clues into how transport happens in a smooth manner. *In vitro* studies have shown that kinesin-1 strictly follows the protofilament axis of microtubules (Ray et al., 1993; Brunnbauer et al., 2012), while dynein and some members of the kinesin superfamily can bind off-axis protofilaments and exhibit more complex motion along the microtubule (Brunnbauer et al., 2012; Vale and Toyoshima, 1988; Valentine et al., 2006; Walker et al., 1990; Yajima and Cross, 2005; Yajima et al., 2008; Pan et al., 2010; Can et al., 2014; Bormuth et al., 2012; Mitra et al., 2015; Mallik et al., 2005; Ross et al., 2006). One hypothesis is that such off-axis motility can be used as a mechanism to bypass obstacles (Ross et al., 2006). However, in living cells it is currently unknown how vesicles carried by multiple motors may circumvent obstacles.

Here, motivated by this question, we used correlative live-cell and multi-color super-resolution microscopy (Bálint et al., 2013; Tam et al., 2014a,b) to show that vesicle size is important for their transport at microtubule intersections. As soon as the intersection spacing is comparable in size to the vesicle, it becomes a steric obstacle leading to vesicle pausing. Development of a new correlative 3D tracking and super-resolution microscopy method allowed us to further study the mechanisms used to circumvent such steric obstacles. Our results show that endolysosomes are transported in two different modes along microtubules and that a small, but significant percentage (∼25%), change their 3D position as they move along the microtubule. Finally, we demonstrated that the 3D repositioning of vesicles on microtubules correlates with the presence of obstacles and may be a mechanism for circumventing them.

## RESULTS

### Microtubules differentially impact trafficking of endolysosomes based on their size

**Fig. 1.**
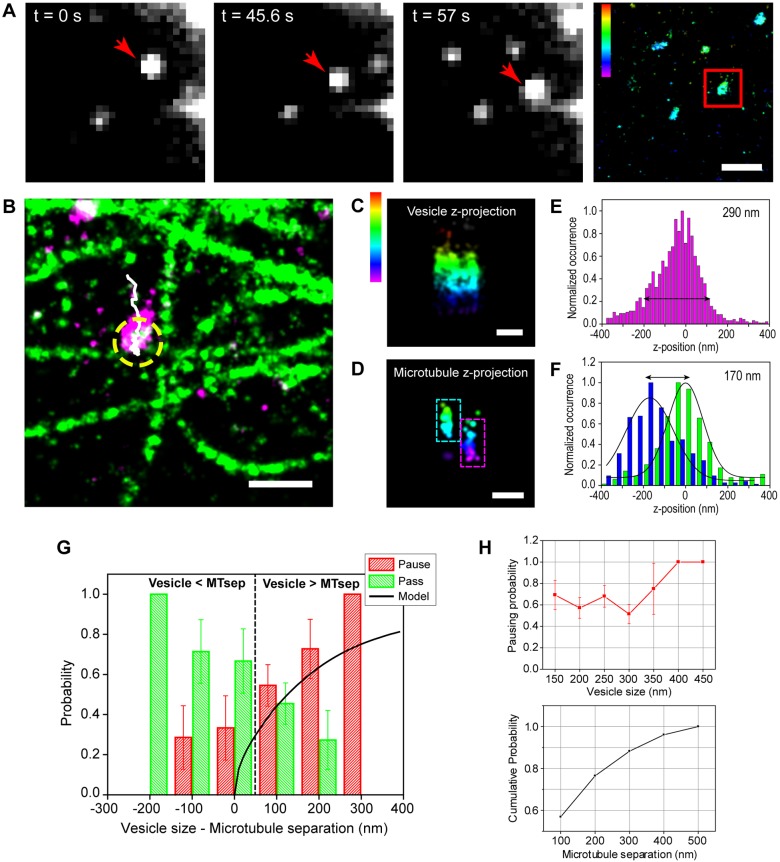
**Vesicles pause at intersections when their size is comparable to microtubule spacing.** (A) Single frames from a live-cell movie of LAMP2–mCherry-labeled endolysosomes. The red arrow indicates the tracked endolysosome, which is also shown in a super-resolution image of the same field of view (red square). (B) A two-color super-resolution image of microtubules (green) and endolysosomes (magenta) after fixation. The trajectory of the vesicle determined by single particle tracking is shown in white. Passive transport is shown with a yellow dotted circle. (C) 3D rendering of the endolysosome and (D) intersecting microtubules. The dashed rectangles show the upper (cyan) and lower microtubule (magenta). (E) Distribution of localizations along the *z*-axis in the super-resolution image of the endolysosome. The diameter of the vesicles was estimated from a pre-established cut-off (black arrow). (F) Distribution of localizations along the *z*-axis in the super-resolution image of microtubules in the region of the intersection. The blue and green plots correspond to the lower and upper microtubules, respectively. (G) Experimental data showing the pausing (red) and passing (green) probability as a function of the difference between endolysosome size and microtubule separation (*n*=51 events, *n*=11 cells). Modeling results are overlaid on top (black curve). (H) Upper plot, pausing probability as a function of vesicle size (*n*=66 vesicles, *n*=11 cells). Lower plot, cumulative distribution of microtubule separations (*n*=51 microtubules, *n*=11 cells). The color scale bar in A,C and D represents the *z*-position (between −400 nm, magenta, and 300 nm, red). Scale bars: 1 µm (A), 500 nm (B), 200 nm (C,D). Error bars (s.d.) in G and H are calculated by bootstrapping.

We previously showed that endolysosomes pass, pause, switch or reverse direction at microtubule intersections (Bálint et al., 2013). An interesting open question is whether trafficking of vesicles with different sizes may be differentially impacted due to the 3D architecture of the microtubule network. To address this question, we carried out correlative live-cell and multi-color super-resolution microscopy to compare the size of endolysosomes to that of microtubule separations (Fig. 1A–F). The microtubule network was stabilized by a low dose of paclitaxel and nocodazole (see Materials and Methods), which was previously shown to not significantly alter vesicle transport as assessed by measuring vesicle speed, pausing frequency, pausing time, run length and processivity, or microtubule post-translational modifications (Bálint et al., 2013). These previous results suggested that the motor–microtubule interactions are not likely to be substantially affected by the experimental conditions, although we cannot fully rule out subtle effects. Further controls verified that microtubules were stable (Fig. S1A,B; see also Bálint et al., 2013). By using polystyrene microspheres internalized in cells as well-defined test structures, we checked that spherical aberrations did not significantly impact the structures imaged in super-resolution, but only led to a slight asymmetry in the images along the *z*-axis (see Materials and Methods; Fig. S1C,E). We also used these microspheres to determine a suitable method for accurately measuring the size of structures in 3D (Fig. S1D,E). As such, we could determine the size of endolysosomes with a resolution below the diffraction limit (Fig. 1C,E; Fig. S1F–H) by measuring the width along the major (Fig. S1G) and minor axis in *xy*-axis, as well as the width along the z-axis (Fig. 1E; Fig. S1H).

The *z*-width determines whether the vesicle can pass through a microtubule intersection. We thus correlated the *z*-diameter of the vesicle with the behavior at intersections (Fig. 1G). These results showed that the pausing probability was low for vesicles smaller than the microtubule separation (Fig. 1G) and went up dramatically when the vesicles became comparable in size to the microtubule separation (Fig. 1G). Interestingly, the majority of microtubule separations are <250 nm leading to a high probability of pausing for vesicles as small as ∼100–150 nm (Fig. 1H).

We next implemented a geometric model in which vesicles of varying sizes were placed at microtubule intersections of varying separations (Fig. S1I). When there was an overlap between the intersecting microtubule and the endolysosome, the vesicle was assumed to pause. For a given vesicle size and microtubule separation, the pausing and passing probability was calculated by determining the range of vesicle positions on the microtubule for which the intersection collides with the vesicle (Fig. S1I). The probability of passing and pausing was then integrated over all possible vesicle sizes weighted by their experimental occurrence (Fig. S1I). The simulation results matched remarkably well with the experimentally determined probabilities for passing and pausing (Fig. 1G). Overall, these results suggest that intersections in the microtubule network act as steric filters to vesicles whose sizes are comparable or larger than the separation of microtubules. Alternatively, excess motors on the vesicle may be able to interact with the intersecting microtubule, leading to the observed pausing. While we cannot fully rule out the latter scenario, we find it less likely as it requires the presence of excess motors at the correct position on the vesicle, yet it has been shown that there are only a small number of motors present on endolysosomal vesicles (Hendricks et al., 2010).

### Endolysosome deformation alone cannot explain how large vesicles pass tight intersections

Previously, we found that pausing at microtubule intersections was temporary and vesicles eventually moved from the intersection after pausing (Bálint et al., 2013). We will refer to the initial behavior at the intersection as ‘encounter’. For those vesicles that initially pause but are able to move once again, we will refer to their behavior after pausing as ‘bypass’ (Bálint et al., 2013). A high percentage of bypass events correspond to the vesicle passing the intersection (Bálint et al., 2013). We hypothesized that those vesicles that eventually passed the intersection could overcome the intersecting microtubule through multiple mechanisms: (1) they could squeeze through the intersection if enough motors engaged to produce high force, (2) they could move around the original microtubule in 3D, and (3) the vesicle could push apart the intersecting microtubules.

**Fig. 2.**
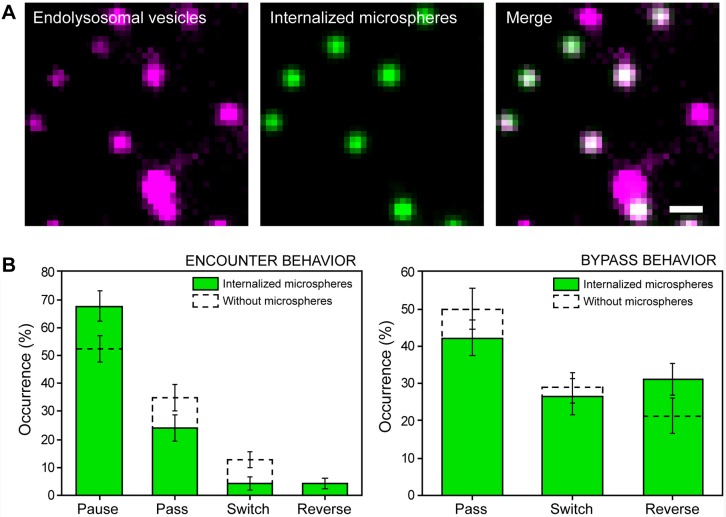
**Endolysosomes containing rigid microspheres exhibit similar encounter and bypass behavior to that shown by native vesicles.** (A) One frame from a live-cell movie of LysoTracker-labeled endolysosomes (magenta) and internalized 450 nm microspheres (green), and an overlay. 94% of motile microspheres were moving inside endolysosomes (*n*=6 cells, *n*=50 microspheres). Scale bar: 2 µm. (B) Encounter (left, *n*=71) and bypass (right, *n*=45) behavior of endolysosomes containing 450 nm microspheres (green) and native LAMP2–mCherry-labeled endolysosomes (dashed lines). Error bars (s.d.) are calculated by bootstrapping.

To test the first mechanism, we internalized 450 nm microspheres into endolysosomes (Fig. 2A; Table S1, Movie 1). In super-resolution images, the center position of the microsphere was aligned with the center position of the vesicle (Fig. S2A), indicating that the microspheres were internalized into the lumen. The rigid microsphere should limit the ability of the vesicle to deform. If shape deformability plays a role, there should be a decrease in the percentage of passing events after pausing. A slightly higher percentage of vesicles containing microspheres initially paused at intersections compared to the proportion of vesicles without microspheres (Fig. 2B, encounter behavior). The pausing period (6.0±9.3 s, *n*=46, mean±s.d.) was comparable to that seen for vesicles without microspheres (6.0±8.0 s, *n*=47, *P*=0.95). After pausing, there was only a small decrease in the passing events (∼10%) (Fig. 2B, bypass behavior). Hence, we conclude that membrane deformation plays only a minor role in overcoming the steric obstacle formed by the microtubule.

### Endolysosomes exhibit complex 3D dynamics along microtubules

To determine whether vesicles move around microtubules, we extended the correlative method to 3D tracking. In principle, off-axis motion should lead to a deviation of the vesicle trajectory perpendicular to the microtubule long axis in 2D. However, since the live-cell movie and the super-resolution image are separated in time, even small changes in the microtubule position during the live-cell movie or fixation can lead to such deviations. Therefore, the 2D trajectory alone is not reliable for determining off-axis motion. The focal plane, on the other hand, constitutes a fixed reference point. Thus, deviations in the *z*-position with respect to the focal plane should not be affected by such artifacts.

**Fig. 3.**
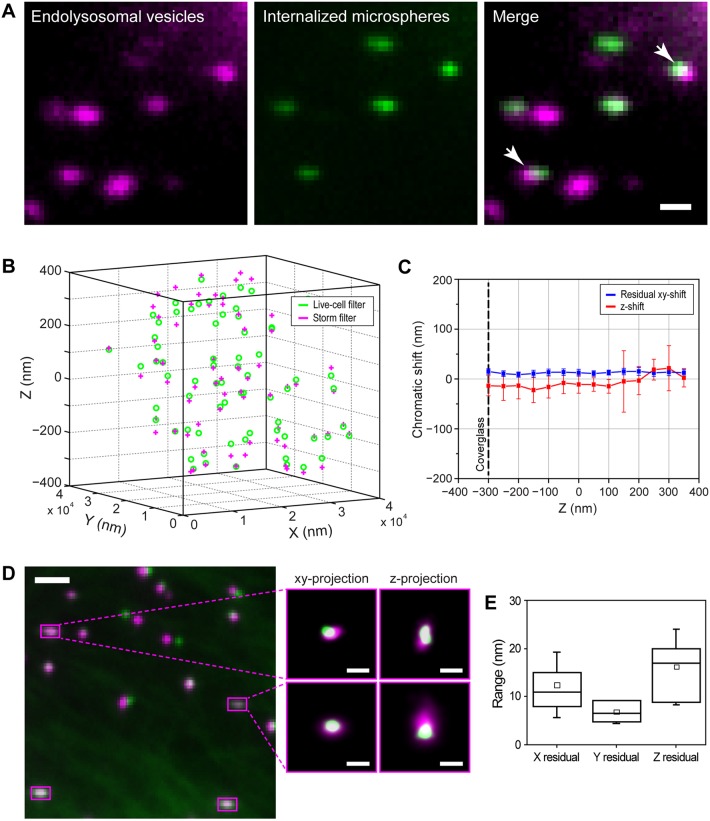
**Internalized microspheres enable correlative 3D tracking and super-resolution microscopy.** (A) One frame from a live-cell movie of LysoTracker-labeled endolysosomes (magenta) and internalized 260 nm microspheres (green), and an overlay. 81% of motile microspheres were moving inside vesicles (*n*=6 cells, *n*=43 microspheres). The arrows show two microspheres that are in different axial planes. (B) Localized 3D position of fluorescent microspheres embedded in a 3D gel matrix. The green circles and pink crosses indicate the localized position in the live-cell and STORM channels, respectively. (C) The raw *z*-shift (red) and the *xy-*shift (blue) after chromatic aberration correction between the localized positions of the microspheres in the two filter sets. The average *z*-shift was –14±5 nm below and 0±14 nm above the focal plane (*n*=698 microspheres). The average residual *xy*-shift after the chromatic aberration correction was 12±2 nm above and 13±1 nm below the focal plane (*n*=698 microspheres). The reported values represent mean±s.d. The black dashed line corresponds to the position of the coverglass. (D) Overlay of the images of the microspheres in the live-cell channel (green) and the super-resolution channel (magenta) after image registration. Microspheres that adsorbed to the coverslip were used as fiduciary markers, which appear to be colocalized in both channels (magenta squares). The zoomed-in images show *xy*- and *z*-projections of the localized center positions of two different reference microspheres on the coverslip after image registration. (E) Boxplot showing the residual registration error in *x*, *y* and *z* calculated as the root mean square distance between the reference microspheres after alignment in different experiments (12±7 nm, 7±2 nm and 16±8 nm in *x*, *y* and *z*, respectively, *n*=15 experiments). The reported values represent mean±s.d. The box represents the 25–75 percentiles. The solid line, the small square and the whiskers are the median, mean and standard deviation, respectively. Scale bars: 1 µm (A), 2 µm (large microsphere images in D), 100 nm (zoomed images in D).

To extend the tracking to 3D, we used a cylindrical lens to introduce astigmatism, which is the same method used to achieve 3D super-resolution imaging (Huang et al., 2008). Since astigmatism relies on engineering the point spread function (PSF) to become elliptical, precise 3D tracking requires a bright and diffraction-limited fluorescent probe that does not intrinsically change its shape. We thus validated 260 nm diameter fluorescent microspheres as effective probes for 3D tracking (Fig. 3A–E; Fig. S2B–I, Table S2, Movie 2) providing a high localization precision in 3D (σ*_x_*=9±4 nm, σ*_y_*=8±4 nm and σ*_z_*=20±9 nm, mean±s.d., *n*=482). With an accuracy of our tracking method of 10–20 nm, it was also necessary to investigate the stability of the microtubules on the nanometer scale, which we achieved by using 3D orbital tracking microscopy. In this method, the laser is orbited about a particle with a radius comparable to the size of the PSF, and the position of a particle is obtained by analyzing the phase and modulation of the intensity distribution along a circular orbit. In combination with an active feedback loop, the orbit of the laser is refocused on the new position of the particle; thereby, the structures in 2D and 3D are tracked with millisecond temporal and nanoscale spatial resolution (Katayama et al., 2009; Lanzano and Gratton, 2012; Dupont and Lamb, 2011) (see Materials and Methods). Since microtubules are linear filaments, any fluctuations should produce deviations in the tracked position in the direction perpendicular to the microtubule long axis. These fluctuations were small, having an average amplitude of 13±4 nm in *x*, *y* and 58±19 nm in *z* (mean±s.d.) on timescales comparable to the duration of active transport trajectories (Materials and Methods and Fig. S2G–I) confirming microtubule stability.

To align the transport trajectories recorded in one color channel (microsphere peak emission of 480 nm) with the super-resolution images obtained in a different color channel (Alexa Fluor 647), we developed a 3D image registration method. By using fluorescent microspheres embedded in 3D inside a gel matrix, we first showed that the chromatic shift in *x* and *y* was constant over the imaged *z*-range (Fig. 3B,C) and could be corrected by using a polynomial transformation (Fig. 3C). Second, the focal shift between the two channels was small and roughly constant over the imaged *z*-range (Fig. 3B,C). By using a polynomial transformation to account for the chromatic shifts and a rigid shift to account for drift, we could align the two images with an accuracy of 12±7 nm, 7±2 nm and 16±8 nm in *x*, *y* and *z*, respectively (mean±s.d.; *n*=15 experiments) (Fig. 3D,E; Materials and Methods).

**Fig. 4.**
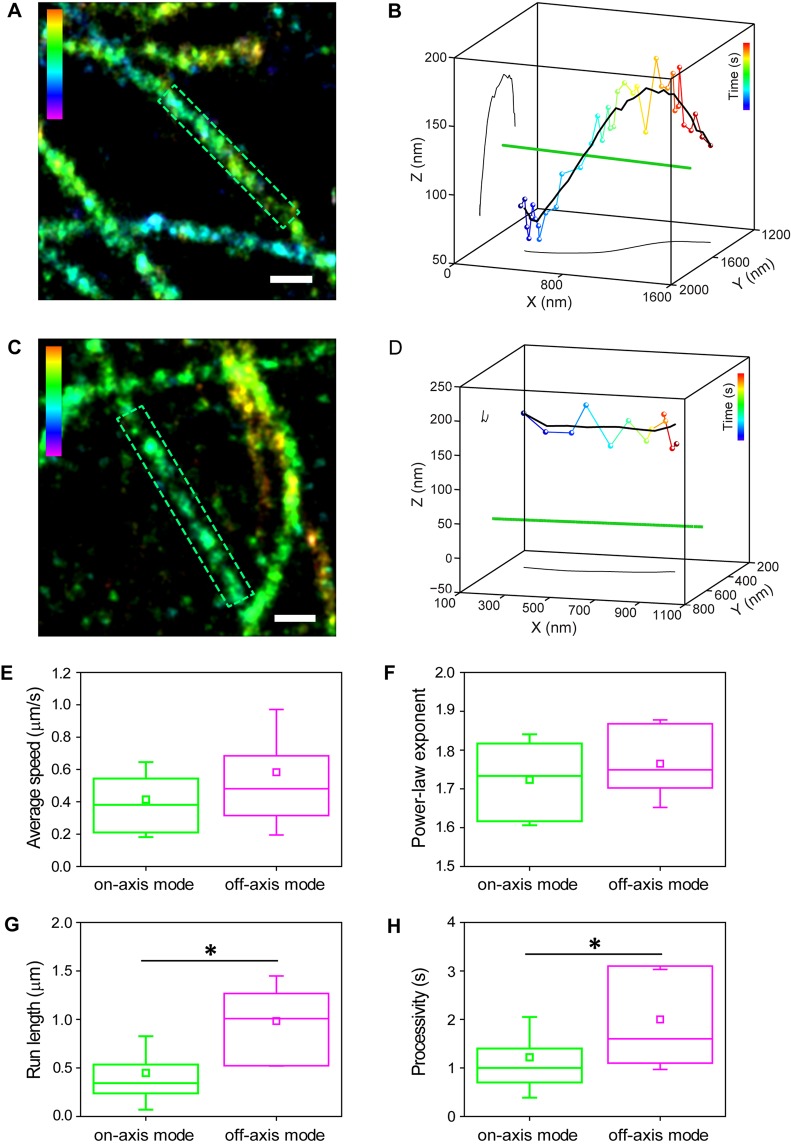
**Vesicles are transported in two separate modes along microtubules.** (A) A 3D super-resolution image of microtubules. The highlighted area defines the microtubule segment where off-axis mode motion was observed. (B) A 3D plot placing the microsphere and endolysosome trajectory in the context of its microtubule path (green, not to scale). The smooth trajectory is shown in black as a 3D plot as well as in an *xy*- and *yz*-projection. (C) A 3D super-resolution image of microtubules. The highlighted area defines the microtubule segment where on-axis mode motion was observed. (D) A 3D plot placing the microsphere and endolysosome trajectory in the context of its microtubule path (green, not to scale). The smooth trajectory is shown in black as a 3D plot as well as in a *xy*- and *yz*-projection. (E–H) Boxplots comparing several motility parameters such as (E) average speed (on-axis mode: 0.4±0.2 µm/s, *n*=42 and off-axis mode: 0.6±0.4 µm/s, *n*=15, *P*=0.05), (F) power-law exponent of the mean square displacement (on-axis mode: 1.7±0.1, *n*=19 and off-axis mode: 1.8±0.1, *n*=12, *P*=0.34), (G) run length (on-axis mode: 0.4±0.4 µm, *n*=42 and off-axis mode: 1.0±0.5 µm, *n*=15, *P*=5×10^−5^) and (H) processivity (on-axis mode: 1.2±0.8 s, *n*=42 and off-axis mode: 2.0±1.0 s, *n*=15, *P*=0.005), for on-axis mode (green)- and off-axis mode (magenta)-type motion. The reported values represent mean±s.d. The box represents the 25–75 percentiles. The solid line, the small square and the whiskers are the median, mean and standard deviation, respectively. *P<0.05 (two-tailed two-sample *t*-test). The color scale bar represents the *z*-position (between −200 nm, magenta, and 350 nm, red) (A), time (between 0 s, blue, and 3.4 s, red) (B), *z*-position (between −400 nm, magenta and 400 nm, red) (C), time (between 0 s blue, and 1.1 s, red) (D). Scale bars: 250 nm.

We first analyzed vesicles moving along isolated microtubule segments (Fig. 4). The average 3D distance between the microsphere center and the microtubule during the active transport phases was 154±61 nm (mean±s.d.; *n*=57 trajectories), which is within the expected range for a microsphere of ∼130 nm radius encapsulated inside a slightly larger vesicle. Each active transport phase of a trajectory was analyzed individually and those subtrajectories that changed their *z*-position by >40 nm with respect to the focal plane (twice the localization precision in *z*) were separated. These subtrajectories were categorized as ‘off-axis mode’ (26%, *n*=15) (Fig. 4A,B) and the remaining subtrajectories (*z*-change of <40 nm) were categorized as ‘on-axis mode’ (74%, *n*=42) (Fig. 4C,D). For the off-axis mode, we further confirmed that the *xy* position for these subtrajectories also underwent similar deviations with respect to the microtubule long axis, suggesting that the vesicle is repositioned in 3D along the microtubule. The change in *x*, *y* and *z* coordinates of the vesicle with respect to the minimum distance to the microtubule during the transport was calculated from the initial and final positions. The average change over multiple off-axis subtrajectories was 83±75 nm in the *xy*-plane and 73±42 nm in the *z*-plane, suggesting that the motion most likely corresponds to partial movement of the vesicle around the microtubule. We also detected these two modes of motion using orbital tracking microscopy (Fig. S3A–D).

We classified off-axis mode transport as anterograde and retrograde based on whether the vesicle was moving away from or towards the cell nucleus and found that it occurred with similar frequency for both directions [7/31 (23%) for anterograde off-axis mode; 7/24 (29%) retrograde off-axis mode]. Often, vesicles underwent transitions between on- and off-axis mode motion (8/15), and both types of motion were observed on the same or adjacent segments of a given microtubule (Fig. S3E–H). In BS-C-1 cells, the main post-translational modifications are detyrosination, tyrosination and acetylation. The majority of microtubules are acetylated, whereas only a small subpopulation is detyrosinated. Thus, we investigated whether the two modes of motion could be due to the presence of a detyrosinated microtubule. Interestingly, two-color super-resolution images of tyrosinated and detyrosinated microtubules, and endolysosomes revealed that the majority of endolysosomes (63%) were associated with detyrosinated microtubules (Fig. S3I) even though detyrosinated microtubules constituted only ∼37% of the total microtubule population (Fig. S3J). We further correlated 3D vesicle trajectories to super-resolution images of detyrosinated tubulin, and scored on-axis mode-like and off-axis mode-like motion from the change in the *z*-position. Since we could not observe all microtubules, we cannot fully determine whether these trajectories occurred along uninterrupted microtubule segments, and thus this is a less strict definition of the transport mode. Nonetheless, vesicles moving along detyrosinated microtubules carried out both on-axis mode (*n*=23 in *n*=5 cells) and off-axis mode-like motion (*n*=18 in *n*=5 cells). Furthermore, vesicles moving in regions that lacked detyrosination signal, and hence likely corresponded to tyrosinated microtubules, similarly carried out both on-axis mode (*n*=26 in *n*=5 cells) and off-axis mode-like motion (*n*=22 in *n*=5 cells). Overall, these results suggest that neither the local properties of microtubules nor their tyrosination state play a major role in dictating the type of motion.

The speed of the vesicles and the power-law exponent of the mean square displacement (MSD) (Materials and methods and Fig. S3K) were similar for both modes (Fig. 4E,F). However, on average, on-axis mode subtrajectories had significantly shorter run lengths (Materials and Methods) than the microtubule segment on which they occurred (median run length of 0.3±0.4 µm and median microtubule length of 1.2±0.6 µm, *n*=42, *P*=3×10^−9^, median±s.d.). Off-axis mode subtrajectories had run lengths that were similar to the length of the microtubule segment (median run length of 1.0±0.5 µm and median microtubule length of 1.5±2.9 µm, *n*=15, *P*=0.09) and these were significantly longer than on-axis mode runs (Fig. 4G, *P*=5×10^−5^). On-axis mode-type motion led to reduced processivity compared to off-axis mode (Fig. 4H). Both modes of active motion were interrupted by passive periods (diffusive and sub-diffusive) with an average power-law exponent of the MSD of 0.9±0.2 (Fig. S3K), which lasted on average 12±13 s for on-axis and 18±23 s for off-axis mode (*P*=0.39). The number of passive events tended to be more frequent for on-axis than for off-axis mode (76% for on-axis mode, *n*=32 and 40% for off-axis mode, *n*=6). Taken together, these results suggest that off-axis mode motion is potentially a more efficient way of moving vesicles.

### Off-axis mode correlates with the presence of roadblocks

**Fig. 5.**
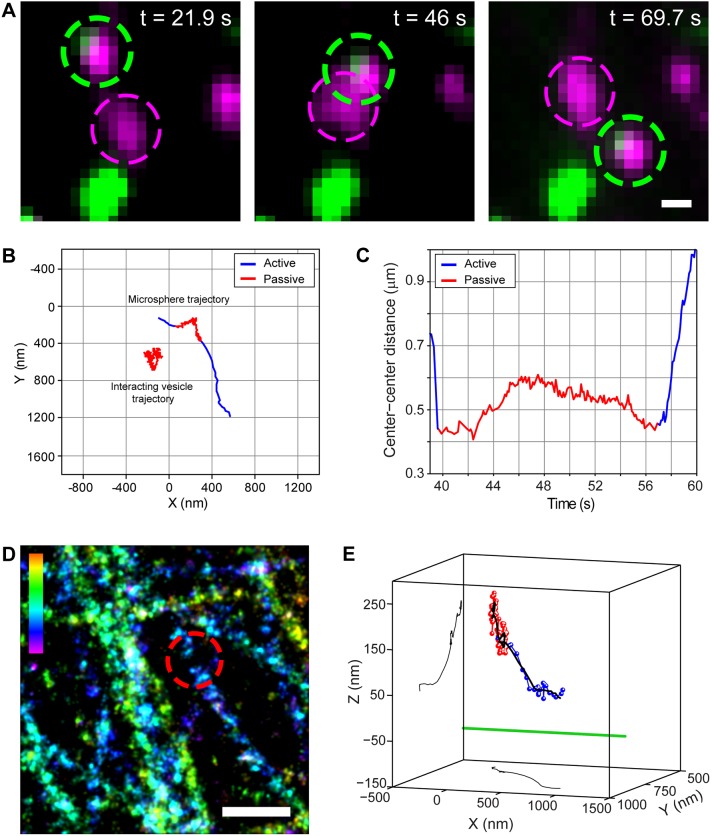
**Off-axis mode motion helps circumvent vesicular obstacles.** (A) Image sequence showing a vesicle–vesicle interaction at different time points. The green and magenta dotted circles highlight the tracked microsphere and the interacting vesicle, respectively. Magenta shows LysoTracker and green shows the microsphere. (B) 2D trajectories of the microsphere-containing and interacting vesicles. (C) The center-to-center distance of the two vesicles before, during and after the encounter. (D) 3D super-resolution image of microtubules. The dotted red circle shows the place of interaction. The color scale bar represents *z*-position (between −200 nm, magenta, and 300 nm, red). (E) 3D plot placing the microsphere trajectory in the context of its microtubule path (green, not to scale). The smoothed trajectory (10-point sliding window average) and its corresponding *xy*- and *yz-*projections are shown in black. For B,C and E, active and passive transport phases in the raw trajectory are shown in blue and red, respectively. Scale bars: 500 nm.

We next analyzed whether off-axis mode correlates with the presence of obstacles by looking at vesicle–vesicle collisions. When one of the vesicles contained a microsphere, we could track its position in 3D (Fig. 5A; Movie 3). In 21/26 cases (*n*=49 cells), the tracked vesicle exhibited passive behavior upon encountering another vesicle. The center-to-center distance of the two vesicles at the closest point of encounter was on average 249±156 nm (*n*=26; mean±s.d.), significantly smaller than the average size of endolysosomes in *xy* (447±145 nm, *n*=106, Fig. S1G), supporting our interpretation that the vesicles are colliding. In 23/26 cases, the tracked vesicle exhibited off-axis mode-like behavior as it moved past the ‘obstructing’ vesicle. For a subset of these cases (*n*=6), we further aligned the trajectory of the vesicle to the microtubule from correlative super-resolution experiments. Fig. 5A and Movie 3 show one such example, in which two vesicles collided, exhibited passive motion (17.3 s) (Fig. 5A–C) and then moved past each other. The vesicle underwent off-axis mode-type motion, showing substantial changes in its 3D position with respect to the microtubule as it moved past the obstructing vesicle (Fig. 5D,E). These changes began during the pausing period and continued during active transport. It is possible that the passive motion is a state in which the motor proteins are not tightly bound to the microtubule, which could potentially aid in switching to off-axis mode transport. This type of positional change was observed for all six cases. Taken together, these observations suggest that off-axis mode-type motion likely aids vesicles in overcoming obstructions formed by other vesicles.

**Fig. 6.**
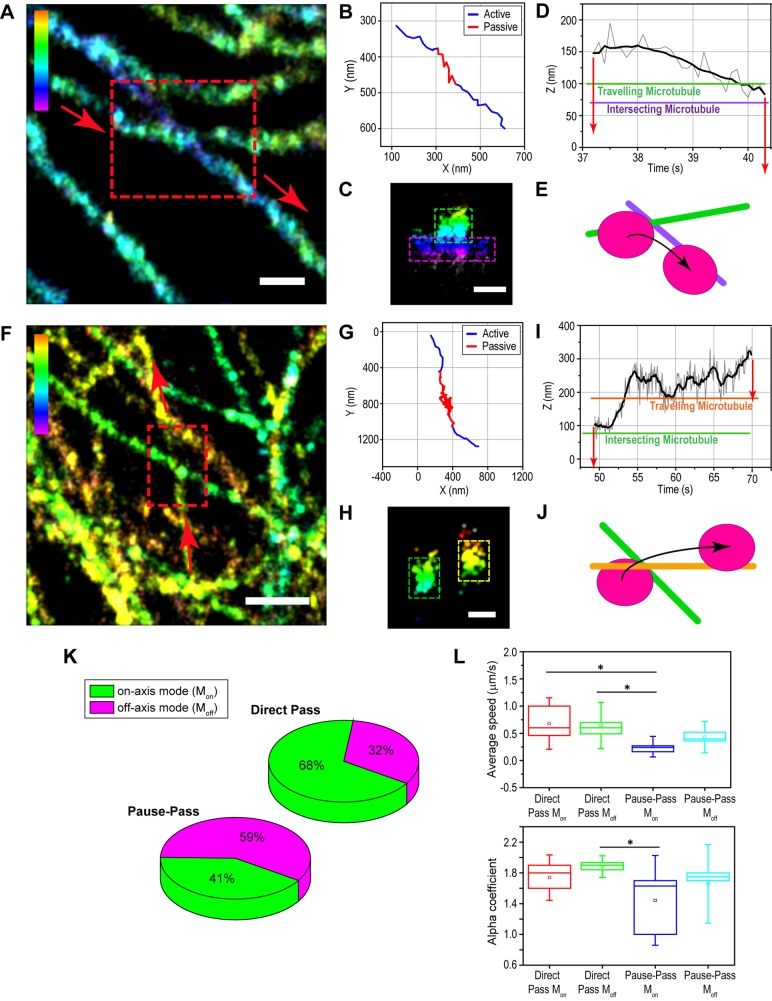
**Off-axis mode motion helps circumvent intersecting microtubule obstacles.** (A,F) A 3D super-resolution image of microtubules. The highlighted area shows the pause-switch (A) and pause-pass (F) events. The arrows indicate the direction of motion. (B,G) 2D trajectory of the tracked endolysosome. Active and passive transport phases are shown in blue and red, respectively. (C,H) 3D projection of the microtubules at the intersection. The green and magenta boxes in (C) and green and yellow boxes in (H) show the upper and lower microtubule, respectively. (D,I) *z*-position of the raw (gray line) and 10-point sliding window average (black line) trajectory as a function of time. The green and purple lines in (D) and orange and green lines in (I) show the *z*-position of the upper and lower microtubule, respectively. The red arrows are an estimate of the radius of the vesicle from the center position at the trajectory. (E,J) Cartoon representation of the pause-switch (E) and pause-pass (J) events. (K) Pie charts showing the percentage of on-axis mode (green) and off-axis mode (magenta) events for endolysosomes that directly passed an intersection without pausing (direct pass, *n*=28) and those that paused before passing (pause-pass, *n*=17). (L) Boxplots for the speeds (upper) for direct pass (on-axis mode: 0.7±0.3 µm/s, *n*=19 and off-axis mode: 0.6±0.3 µm/s, *n*=9) and pause-pass (on-axis mode: 0.3±0.1 µm/s, *n*=7 and off-axis mode: 0.4±0.2 µm/s, *n*=10), *P*=0.005 between pass off-axis mode and pause-pass on-axis mode and *P*=0.002 between pass on-axis mode and pause-pass on-axis mode; and the power law exponent (lower) for direct pass (on-axis mode: 1.7±0.2, *n*=17 and off-axis mode: 1.9±0.1, *n*=8) and pause-pass (on-axis mode: 1.4±0.4, *n*=6 and off-axis mode: 1.7±0.3, *n*=6), *P*=0.009 between pass off-axis mode and pause-pass on-axis mode. The reported values represent mean±s.d. The box represents the 25–75 percentiles. The solid line, the small square and the whiskers are the median, mean and standard deviation, respectively. The *P*-values are calculated for a two-tailed two-sample *t*-test and (*P<0.05). The color scale bar represents the *z*-position (between −100 nm, magenta, and 350 nm, red, in A; −400 nm, magenta, and 400 nm, red, in F). Scale bars: 250 nm (A,C), 500 nm (F), 200 nm (H).

Next, we analyzed switching events at microtubule intersections. Fig. 6A–E shows one example in which the two microtubules were separated by <100 nm, and the vesicle was initially on the upper microtubule. At the intersection, the vesicle paused and switched to the lower microtubule (Fig. 6D,E). During the switch, the *z*-position of the vesicle decreased by 75 nm as it moved from the upper to the lower microtubule (Fig. 6D). We frequently observed such changes in the *z*-position of endolysosomes as they switched microtubules at intersections (*n*=6/10 cases). Similar *z*-changes were also observed in orbital tracking experiments (Fig. S4).

Finally, we investigated whether similar mechanisms played a role when pausing vesicles eventually passed a tight intersection (Fig. 6F–J). Fig. 6F–J shows one example in which a vesicle that was initially sterically hindered by an intersecting microtubule changed its *z*-position by 200 nm to overcome the obstacle. For those vesicles that passed a microtubule intersection without any pausing, 68% followed the on-axis mode transport (*n*=19) whereas 32% followed off-axis mode transport (*n*=9) (Fig. 6K). It is possible that in cases where off-axis mode was observed, that there were other obstacles along the microtubule. Alternatively, a switch to an off-axis mode may sometimes happen stochastically. For those vesicles that paused at intersections, a much higher percentage (59%, 10/17) underwent a substantial change in their 3D position with respect to the microtubule (Fig. 6K). These 3D changes either started during the active period of motion after pausing (2/10), similar to off-axis mode transport or during the pausing period (8/10). Interestingly, not all vesicles that paused at intersections repositioned to pass the intersection. In these cases, it is possible that the vesicle can push apart the two microtubules or the two microtubules can dynamically change their *z*-separation. The latter scenario is less likely since the orbital tracking experiments showed that microtubules were stable. In support of the former scenario, the pausing time of these vesicles was slightly longer than those that passed by changing their 3D position (5.9±5.4 s, *n*=7 versus 3.8±3.4 s, *n*=10, respectively). In addition, these vesicles had a tendency to pass the intersection with lower speed and slightly decreased power-law exponent of the MSD (Fig. 6L), suggesting that they had a harder time passing through the intersection.

## DISCUSSION

### Cytoskeletal impact on transport

Transport is regulated by multiple mechanisms, yet the impact of the local cytoskeletal structure on transport has only been poorly explored. The microtubule network of most cells is highly dense leading to a large number of intersecting microtubules. A vesicle traveling between the cell periphery and the nucleus (a distance of several tens of microns) encounters, on average, one or two intersections for every micron of its trajectory, or tens of intersections in total. Short pausing events at such ‘roadblocks’ can play an important physiological role as long as mechanisms are in place to avoid permanent stoppage. Longer pauses can limit cargo delivery between the cell periphery and the perinuclear region, as well as provide additional hindrances for other organelles by blocking the intersection. Previously, it has been shown that endosome pausing correlates with regions of high microtubule density and endosomes can undergo fission events during pausing (Zajac et al., 2013). These events may be important for sorting recycling cargos from those destined for degradation (Zajac et al., 2013). Modeling work has suggested that microtubule switching at intersections may be regulated by microtubule spacing and motor number (Erickson et al., 2013). Our previous work has demonstrated that endolysosomes pause at tight intersections (Bálint et al., 2013). Here, using live-cell and multi-color super-resolution microscopy, we further showed that intersections lead to pausing of vesicles that are comparable in size to the separation of microtubules at the intersection. This pausing may be due to a steric effect of the intersecting microtubule or molecular interactions between unbound motors on the vesicle and the intersecting microtubule. The fact that vesicles started pausing as soon as their size was comparable to the intersection spacing suggests that there is not enough force exerted by the translocating motors to deform the membrane of the vesicle. These results are consistent with previous studies that showed that the membranes of endosomes are under high tension and endosomes can deform the ER network (Zajac et al., 2013).

In the future, it would be interesting to more directly test the impact of these intersections both on physiological events such as fusion and fission, as well as transport efficiency. This could be investigated by reducing microtubule density using microtubule-depolymerizing agents or by using photoinducible crosslinkers attached to motor proteins to initiate stalling of vesicles at intersections.

### Off-axis mode correlates with presence of roadblocks

The mechanisms utilized when circumventing barriers are unclear. Our correlative experiments revealed that, in ∼25% of the cases, vesicles exhibited substantial 3D positional changes as they were actively transported along microtubules (off-axis mode motion). Off-axis mode motion is not likely to correspond to differences in microtubule protofilament number, since most microtubules contain 13 protofilaments *in vivo* (Amos and Schlieper, 2005; Tilney et al., 1973) and have a protofilament axis aligned with the microtubule long axis. The two modes of motion were also independent of the tyrosination and detyrosination of microtubules.

The 3D motion observed was generally consistent with a partial rotation around the microtubule and could be due to several mechanisms. For example, single motors may side-step to off-axis protofilaments. Several *in vitro* experiments have demonstrated that the retrograde motor dynein can indeed side-step to off-axis protofilaments (Can et al., 2014; Mallik et al., 2005; Ross et al., 2006; Mitra et al., 2015; Vale and Toyoshima, 1988), which is consistent with our observations of off-axis mode motion in the retrograde direction. Kinesin-1 strictly follows the protofilament axis of the microtubule (Brunnbauer et al., 2012; Ray et al., 1993), whereas members of the kinesin-2 subfamily exhibit a high variability (Brunnbauer et al., 2012). Endolysosomal vesicles are associated with both kinesin-1 and kinesin-2 motors (Hendricks et al., 2010; Maday et al., 2014). Thus, the anterograde off-axis mode motion could be due to kinesin-2 being more active during off-axis mode transport. Indeed, kinesin-2 has been shown to be more robust against detachment when encountering obstacles (Hoeprich et al., 2014). Recent *in vitro* experiments showed that kinesin-1 can also bind to neighboring protofilaments in order to circumvent permanent roadblocks (Schneider et al., 2015). Thus, it is possible that motors can change their mechanism of motion in the presence of obstacles. Alternatively, off-axis motion could also be the result of cooperation or even a tug of war involving the detachment of one motor and binding of another motor to neighboring protofilaments. The small mesh size of the actin and microtubule network limits the ability of large vesicles to diffuse away from the microtubule during such detachment events and allows the motors to readily bind back to the microtubule, which can help motors explore the full 3D microtubule surface. Yet another possibility is that multiple motors may walk on different protofilaments during vesicle transport leading to a rolling of cargo around microtubules. Finally, the vesicle itself may bend in 3D away from obstacles as it moves past the obstacle while the motors may remain bound to the same protofilament. In this case, we would expect the vesicle to return to its original position with respect to the microtubule after passing the obstacle, which we do not observe. In the future, knocking down of motors or recruitment of specific motors with varying copy numbers to the vesicles using optogenetic approaches (Ballister et al., 2015; van Bergeijk et al., 2015) may help dissect the contribution of these mechanisms to off-axis mode transport.

## MATERIALS AND METHODS

### Cell culture

A stable cell line for GFP–tubulin was derived from African green monkey (*Cercopithecus aethiops*) kidney epithelial cells (BS-C-1, American Type Culture Collection, ATCC, CCL-26). Cell culture consisted of complete growth medium [Minimum Essential Medium Eagle with Earle's salts and nonessential amino acids plus 10% (v/v) fetal bovine serum (FBS), 2 mM L-glutamine and 1 mM sodium pyruvate; 500 µg/ml of Geneticin^®^ (G418 Sulfate) and penicillin-streptomycin] at 37°C and 5% CO_2_.

A double stably transfected cell line for GFP–tubulin and LAMP2–mCherry (Lgp120NL–mCherry) was derived from the BS-C-1 GFP–tubulin cell line following pHyg-LGP120NL-mCherry plasmid transfection and clone selection. Cell culture medium included an additional 100 µg/ml Hygromycin B (Invitrogen 10687010).

Cells were plated on fiduciary marker (carboxyl fluorescent 260 nm yellow microspheres or carboxyl fluorescent Nile Red 240 nm microspheres, Spherotech) and fibronectin (20 µg/ml)-coated eight-well Lab-Tek 1 coverglass chamber (Nunc). Paclitaxel, nocodazole and other chemicals were purchased from Sigma-Aldrich. Cell culture medium and additives were purchased from GIBCO (Life Technologies).

### Microsphere internalization

Cells were incubated with the 260 nm or 450 nm diameter carboxyl fluorescent yellow microspheres (Spherotech) at a 1:100 dilution in complete growth medium for 30 min at 4°C and 30 min at 37°C. Cells were washed with fresh medium and incubated at 37°C for 60 min (260 nm microspheres) or overnight (450 nm microspheres). The 450 nm diameter biotinylated microspheres (Spherotech) were also internalized into cells by overnight incubation (1:20 dilution in complete growth medium) at 37°C.

### Paclitaxel and nocodazole treatment of cells

Cells were pre-treated with 120 nM paclitaxel and 120 nM nocodazole in complete growth medium for 10 min at 37°C and subsequently maintained with these drugs at 24°C for the duration of the experiment (∼45 min).

### Endolysosomal vesicle labeling for live-cell imaging

Endolysosomes were incubated with LysoTracker^®^ Red DND-99 (Invitrogen) at a concentration of 50 nM for 10 min at 37°C during the paclitaxel–nocodazole pre-treatment.

### Immunostaining

Cells were fixed and immunostained as described in Bálint et al. (2013). Briefly, fixation was performed with 3% (v/v) paraformaldehyde and 0.1% glutaraldehyde in PBS. A 0.1% NaBH_4_ solution in PBS was used to quench background fluorescence. Cells were blocked with 3% (w/v) bovine serum albumin (BSA) and 0.2% Triton X-100 (Fisher Scientific) (v/v) in PBS, and incubated with the appropriate dilution of primary and secondary antibodies in the same blocking buffer. Cells were rinsed with washing buffer [0.2% BSA, 0.05% TritonX-100 (Fisher Scientific)] between antibody incubations.

The primary antibodies used were: rabbit polyclonal (YL1/2) to α-tubulin (Abcam, ab18251), at a dilution of 1:150; rabbit polyclonal (YL1/2) to detyrosinated tubulin (Abcam, ab48389), at a dilution of 1:100; rat monoclonal to tyrosinated tubulin (Millipore, mab1864-1), at a dilution of 1:100 and chicken polyclonal (YL1/2) to mCherry (Novus Biologicals, NBP2-25158. Lot 6695), at a dilution of 1:500. The secondary antibodies used were: AffiniPure Donkey anti-Rabbit IgG (H+L) (Jackson ImmunoResearch, 711-005-152) at a dilution of 1:100, and AffiniPure Donkey anti-Rat IgG (H+L) (Jackson ImmunoResearch, 712-005-150) at a dilution of 1:100 and AffiniPure Donkey anti-Chicken IgY (IgG) (H+L) (Jackson ImmunoResearch, 703-005-155) at a dilution of 1:100. For stochastic optical reconstruction microscopy (STORM), the secondary antibodies were labeled with an Alexa Fluor 405 and Alexa Fluor 647, or a Cy3 and Alexa Fluor 647 activator–reporter dye pair combination at concentrations of 0.12–0.15 mg/ml (Bates et al., 2007).

After internalization of 450 nm biotinylated microspheres (Spherotech), cells were fixed and blocked using the previously described protocol. Cells were incubated for 40 min with a 1:20 dilution of Alexa Fluor 405 and Alexa Fluor 647-labeled streptavidin (0.2 mg/ml) and washed with PBS.

### Live-cell imaging

Live-cell imaging was carried out with a custom-built wide-field fluorescence microscope as described previously (Bálint et al., 2013). Imaging was performed at 24°C. Dual-color live-cell imaging was performed by using two laser sources: 488 nm from an argon-krypton laser (Spectrum IC70, Coherent) for exciting GFP–tubulin and the internalized microspheres, and a 560-nm fiber laser (MPB Communications) for exciting LysoTracker Red DND-99 (Invitrogen) or LAMP2–mCherry. The emitted fluorescence was split by a quad band set (TRF89902-ET-405/488/561/647 Laser Quad Band Set for TIRF applications, Chroma Technology) for the 3D tracking experiments or by two separate emission filters (ET525/50 and ET605/52, Chroma Technology) for the rest of the experiments. A 1-m focal length cylindrical lens was inserted in front of the camera for 3D imaging (Huang et al., 2008). The exposure time in the dual-color live-cell imaging experiments was 100 ms or 50 ms (effective exposure time of 200 ms or 100 ms). To keep the sample in focus, a home-built focus lock system was used (Bates et al., 2007).

### Confocal imaging

Confocal movies of the microtubule network in drug-treated living cells were acquired at 2 s/frame. An 800-nm *z*-range was scanned in 125 nm steps and the duration of a full *z*-stack acquisition was 20 s.

### STORM imaging

STORM was acquired with the same custom-built microscope. Laser light at 647 nm from an argon-krypton laser (Spectrum IC70, Coherent) was used for exciting Alexa Fluor 647 (Invitrogen) and a 405-nm solid-state laser (Cube, Coherent) was used for reactivating the Alexa Fluor 647 (Invitrogen) via an activator dye (Alexa Fluor 405). The emitted light from Alexa Fluor 647 (Invitrogen) was collected by the objective, filtered by an emission filter (ET705/72m, Chroma) and imaged onto the EM-CCD camera at 20 ms per frame.

### Orbital tracking

Orbital tracking experiments were performed on an upgraded version of the home-built confocal system described previously (Dupont et al., 2013) using two galvanometer mirrors and a self-written code (LabVIEW) running on a field-programmable gate array combined with a real-time processor (cRIO-9082, National Instruments). In this configuration, we achieved a spatial resolution of <5 nm in *x* and *y* and <30 nm in *z* with a temporal resolution of <5 ms (at a count rate of >80 kHz per channel).

### Single-particle tracking with the orbital-tracking microscope

Orbital tracking in BS-C-1 cells stably expressing GFP–tubulin and LAMP2–mCherry was performed at 25°C under paclitaxel–nocodazole treatment, at a 5 ms time resolution and a laser power of <10 µW (561 nm) before entering the objective lens for LAMP2–mCherry endolysosome tracking and <10 µW (488 nm) before the objective for imaging of the GFP-tubulin microtubules.

### GFP–tubulin tracking

3D orbital tracking can be used to determine the 3D position of an elongated structure, e.g. microtubules, with nanoscale precision. By using fluorophores that are susceptible to photobleaching, the orbit will burn a ‘hole’ in the fluorescent signal emitted by the elongated structure. Hence, the tracking algorithm always re-centers the orbit to the brightest signal in the vicinity. This leads to the orbit following the path of the 3D elongated structure.

### Data analysis

#### Image registration

Channel registration between the live-cell movies of internalized microspheres acquired using the quad band filter set (GFP emission) and the super-resolution images acquired with the STORM filter set (Alexa Fluor 647 emission) was performed in two steps. First, a 2D second-order polynomial transformation was calculated based on the position of fiduciary markers (carboxyl fluorescent yellow microspheres 260 nm, Spherotech) visible in both channels to correct for chromatic aberrations. 2D chromatic aberrations between the two channels were constant throughout all *z*-planes. Thus, it was sufficient to apply a 2D second*-*order polynomial transformation to the super-resolution raw localizations within the whole imaged *z*-range. As a second step, to account for sample drift between the live-cell and the super-resolution imaging, we calculated a rigid shift in *x*, *y* and *z* by using fiduciary markers on the glass surface of the samples that were visible both in the live-cell movie and the super-resolution image (*n*=4–7 fiduciary markers). The rigid shift was applied to the super-resolution raw localizations after the 2D second-order polynomial transformation. The alignment precision was calculated as the root mean square difference in the aligned position of the fiduciary markers present in the sample in the *x*, *y* and *z* positions given by

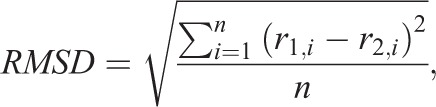

where *r* represents *x*, *y* or *z* coordinates; *1* and *2* correspond to the two channels to be aligned; *i* corresponds to the fiduciary marker and *n* corresponds to the total number of fiduciary markers.

A similar two-step procedure was applied for channel registration of LAMP2–mCherry vesicles or 450 nm microspheres in live-cell movies acquired using the ET605/52 or ET525/50 emission filters (Chroma Technology), respectively, and the corresponding super-resolution images acquired with the STORM filter set (Alexa Fluor^®^ 647 emission). A 2D affine transformation was calculated from fiduciary markers (carboxyl Nile Red microspheres 240 nm, Spherotech) visible in both channels and applied to the live-cell movies by means of the MultiStackReg plug-in of ImageJ (National Institutes of Health). Drift between the live-cell experiments and the super-resolution imaging was corrected by applying a rigid shift in *x* and *y*.

#### 3D single-particle tracking

Endolysosomal vesicle and microsphere positions were tracked by a semi-automated custom-written particle tracking software capable of performing 2D and 3D single-particle tracking. To determine the *x* and *y* positions, 2D or 3D trajectories were analyzed by performing a 2D Gaussian fit or an elliptical Gaussian fit to the object PSF, respectively. Additionally, the *z*-positions were determined by comparing the widths of the PSF in *x* and *y* to a predetermined calibration curve obtained using the particular live-cell filter set. The calibration curve was determined by imaging 260 nm carboxyl fluorescent yellow microspheres (Sperotech) on glass at different focal planes achieved by means of a piezoelectric *z*-stage (NanoScanZ Prior Scientific). The imaging depth was within 200–300 nm of the glass surface. Sample drift during acquisition was calculated by tracking the *x*, *y* and *z* position of the fiduciary markers adsorbed onto the glass surface and subsequently subtracted from the trajectories.

A custom-written MATLAB script was used to determine active and passive phases from the 2D trajectory information. We performed a moving window analysis (four-point segments) along the trajectory data points. We then calculated the ratio between the total displacement between the initial and final points of the segment and the sum of displacements between the points within the segment. Because the segments overlap, each point within a trajectory will appear in multiple segments. Thus, for every data point, the ratios were averaged over all segments containing this specific point. This ratio estimates the linearity of each segment; hence, values close to 1 correspond to active phases. A threshold ratio of 0.8 was chosen to distinguish between active and passive phases. Additionally, we used an angle criterion (successive displacement vectors showing angles less than 90° are categorized as passive) to further filter the initial categorization. The chosen parameters were optimized by visual inspection and comparison of a select number of trajectories (*n*=10) to a published method based on a Hidden Markov Model (HMM) analysis (Monnier et al., 2015). After this analysis, we considered the segments with >12 data points and calculated the power-law exponent from the MSD to further confirm the previous categorization. The exponents were calculated by fitting the MSD curves to the power-law *log*[*MSD*(Δ*t*)]=*α*  · *log*(Δ*t*)+*C*. Active transport was separated from passive transport based on *α*>1.5. In all cases, the determined power-law exponent matched our initial categorization, confirming that this analysis was effective. Next, we considered the segments of trajectories with <12 data points, for which MSD could not be calculated with high confidence. The segments initially categorized as passive and containing <5 data points (500 ms) were considered to be inconclusive and were merged with the segment before and after them. The segments initially categorized as active with a total displacement <160 nm (one pixel) were categorized as passive, and the rest as active. The displacement (160 nm) was chosen based on the fact that the vesicles on average move at speeds of ∼0.4 µm/s and, in five frames (500 ms), the expected displacement is ∼200 nm. Additional parameters such as run length, processivity and average speed were also computed using the same custom-written program after the active or passive categorization of the trajectories.

Endolysosome–microsphere trajectory pairs were analyzed with a custom-written MATLAB script. Correlation coefficients for endolysosome–microsphere *x* and *y* positions were computed with the MATLAB function *corrcoef* which calculates the coefficients as:
(1)
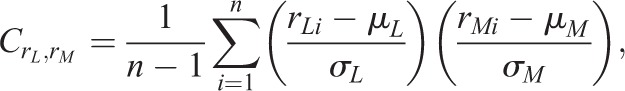
where *r* represents *x*, *y* position; *L* and *M* represent the vesicles and its corresponding internalized microsphere, respectively; *σ* represents the standard deviation of the position over the entire trajectory; *µ* represents the mean position over the entire trajectory; and *n* represents the total number of trajectory data points.

#### 3D STORM data analysis

STORM images were analyzed and rendered as previously described (Huang et al., 2008) using custom-written software (Insight3, kindly provided by Bo Huang, Department of Pharmaceutical Chemistry and Department of Biochemistry and Biophysics, UCSF, CA, USA). Spectral cross-talk in two-color experiments was subtracted as previously described (Dani et al., 2010).

Microtubule separation was measured as described in Bálint et al. (2013). The microtubule position was extracted from the super-resolution raw localizations by first removing outlier localizations more than one standard deviation away from the mean *z*-position of all the localizations. The remaining localizations were smoothed by averaging over one-tenth of the total number of localizations in that particular super-resolution image of the microtubule. A three-dimensional linear fit was performed on the smoothed localizations to extract the microtubule position.

The percentage of microtubule area for detyrosinated versus tyrosinated tubulin was calculated by using ImageJ (National Institutes of Health) and estimating the total pixel area from a binary image of microtubules generated from a sequential, two-color super-resolution image. The number of endolysosomes associated with detyrosinated tubulin was calculated by manually counting the vesicles overlapping with detyrosinated microtubules. The total number of endolysosomes was obtained by using the ‘Analyze Particles’ tool from ImageJ.

The standard deviation in *x*, *y* and *z* (σ*_x_*, σ*_y_* and σ*_z_*) of the 3D images of 450 nm biotinylated microspheres internalized into cells showed that σ*_x_*=σ*_y_*, whereas σ_z_ was 3% larger for *z*<100 nm and 5% smaller for *z*>100 nm than σ*_x_* and σ*_y_*, demonstrating that the impact of spherical aberrations at the imaging depth (200–300 nm) was small. A threshold of 20% of the localizations contained in the plane with the maximum number of localizations was set to determine the size of the microspheres in 3D.

#### Bootstrapping error estimates

Error estimates in the form of standard deviations obtained via bootstrapping were determined by randomly re-sampling from the original data with replacement (over 100 iterations) being the re-sampled data the size of the original data.
